# Fecal microbiota landscape of commercial poultry farms in Faisalabad, Pakistan: A 16S rRNA gene-based metagenomics study

**DOI:** 10.1016/j.psj.2025.105089

**Published:** 2025-03-23

**Authors:** Muhammad Moman Khan, Muhammad Ahmed Mushtaq, Muhammad Suleman, Umer Ahmed, Muhammad Faisal Ashraf, Rizwan Aslam, Mashkoor Mohsin, Stefan Rödiger, Yasra Sarwar, Peter Schierack, Aamir Ali

**Affiliations:** a Institute for Biotechnology, Brandenburg University of Technology (BTU) Cottbus-Senftenberg, Senftenberg, Germany; b Clemson University, Clemson, United States; c Institute of Microbiology, University of Agriculture, Faisalabad, Pakistan; d National Institute for Biotechnology and Genetic Engineering College, Pakistan Institute of Engineering and Applied Sciences (NIBGE-C, PIEAS), Faisalabad, Pakistan

**Keywords:** 16S rRNA, Broiler, Farm-type, Layer, Metagenomics

## Abstract

This study explores the microbiota of broiler and layer farms, aiming to understand how genetic breed, age, and farm type influence microbial communities in commercial settings. Fecal samples from 18 poultry farms (twelve layers and six broilers) in Faisalabad, Pakistan were analyzed using 16S rRNA gene sequencing of the V3-V4 region to evaluate bacterial composition. The dominant phylum, *Firmicutes*, accounted for 58.72 % of the microbial population, with *Lactobacillus* being the most abundant genus in both broilers and layers. The total abundance of potentially pathogenic genera was also assessed with *Enterococcus* and *Corynebacterium* being the most prevalent across all farms, regardless of bird type. Layers exhibited greater microbial richness and diversity than broilers, while the Karachi cage system (KCS) farm type showed higher richness than Floor system (FS). Although the breed significantly influenced microbial diversity, age was not a determining factor. Co-occurrence analyses revealed close interactions among phyla (*Actinobacteriota, Proteobacteria, Firmicutes, Fusobacteriota*, and *Bacteroidota*) and genera (*Lactobacillus, Brevibacterium, Enterococcus*), suggesting their pivotal roles within the microbial community. Additionally, functional analysis detected important metabolic pathways and traced microbial signatures of key pathogenic bacteria, enhancing our understanding of microbial contributions to poultry health. Despite limitations such as the need for broader geographic sampling and accounting for diet and medication, this study advances microbiome research in Pakistan's poultry sector, emphasizing consistent taxa and opening avenues for future investigations into microbiome manipulations for improved food safety and achieve better sustainable practices.

## Introduction

Worldwide consumption of poultry meat steadily increased over the last few decades and is predicted to increase even more in the future (OECD, 2020). To meet the food requirements of the growing human population, it is important that poultry meat and eggs are easily accessible and affordable for the general populace as sources of high-quality protein. In order to accommodate the increasing demand, the poultry industry is continuously increasing production efficiency (Dehau et al., 2022). During the last six to seven decades, an intensive selective breeding has been conducted to enhance industrial output of chickens that utilizes the feed efficiently and produce adequate muscle-mass to meet the nutritional requirements. In Pakistan, poultry sector is an integral part of the agricultural economy and contributes 1.3 % of the gross domestic product. Industrial poultry farming began in the 1960s and has since evolved to supply a significant portion of the population's daily protein intake (Hussain et al., 2015). Pakistan is the 11th largest poultry producer in the world annually, contributing around 40.7 percent of the country's gross meat production (“Pakistan Economic Survey,” 2023). With thousands of farms spread across the country, poultry is the second largest industry economically, and the leading commercial broiler strains are Cobb, Ross, Arboracre and Hubbard (Chand et al., 2018; Nisar et al., 2019).

Efficient energy and nutrient conversion from feed depends on interactions between the chicken's metabolic functions and the microbiome in its gastrointestinal tract, where the bacterial community directly impacts flock health, production, and immune system (Clavijo and Flórez, 2018). Over the past decade, microbiota research has transformed our understanding of aspects affecting poultry health and production (Dehau et al., 2022). The symbiotic relationship between the host and commensal microbiota is closely related to the host's environment (Qi et al., 2019; Malard et al., 2021) and serves as an important metabolic organ (Wen et al., 2019), influencing the feed conversion ratio (Cui et al., 2017), nutrient uptake (Pan and Yu, 2014), immunity (Yeoman et al., 2012), and pathogen resistance (Clavijo and Flórez, 2018). Microbial abundance and diversity vary by intestinal segment, playing critical roles in maintaining intestinal integrity. Thus, understanding of the intestinal microbiota composition in laying hens and broilers is essential for health management (Alvarenga et al., 2023).

This study investigates the fecal microbiota of poultry from 18 commercial farms in Faisalabad, Pakistan, encompassing both layer and broiler chickens raised under varying conditions of farming systems, bird ages, and breeds. The primary objective was to explore the compositional differences in the microbial communities and assess the impact of these variables on microbiota diversity. Furthermore, the study aimed to identify dominant taxa, detect potentially pathogenic genera, predict bacterial co-occurrence networks, and mapped the inferred functions of the microbial community.

## Materials and methods

### Sample collection and DNA extraction

**Fig. 1:**
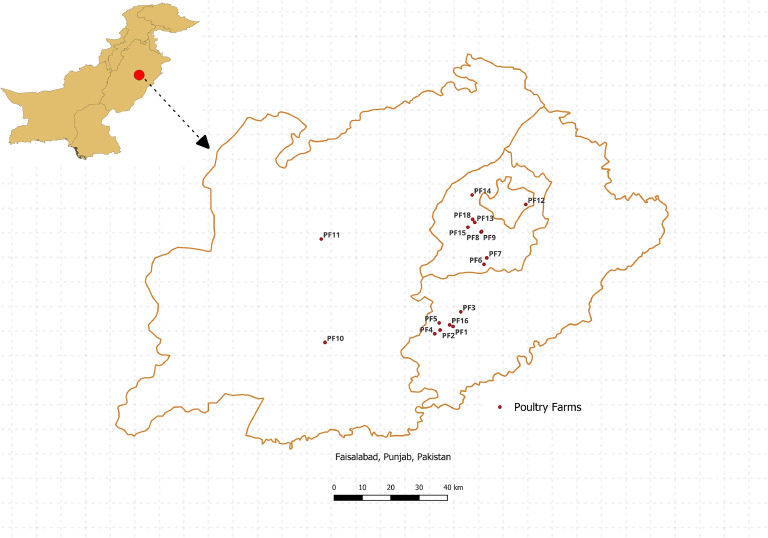
Map of Faisalabad, Pakistan, depicting the sample locations of different commercial poultry farms.

Fresh fecal samples were collected by sterile forceps from 18 different commercial farms: broilers (*n* = 6) and layer (*n* = 12) across the district of Faisalabad, Pakistan, as detailed in Table 1 and the locations are depicted in Fig. 1. The farm types are explained in detail in Supplementary file-1. Three fecal samples per farm (exception two fecal samples from poultry farm number 16) were collected aseptically, placed into sterile tubes, and stored at −20°C until required.

**Table 1:** List of Samples.

Sample	Farm	Bird type	Age	Breed⁎⁎	Farm type*
1	Poultry Farm 1	Layer	16 Weeks	WC36	KCS
2					
3					
4	Poultry Farm 2	Layer	28 Weeks	LSL	FS
5					
6					
7	Poultry Farm 3	Layer	88 Weeks	CNC	KCS
8					
9					
10	Poultry Farm 4	Layer	16 Weeks	WC36	FS
11					
12					
13	Poultry Farm 5	Layer	60 Weeks	CNC	FS
14					
15					
16	Poultry Farm 6	Layer	30 Weeks	LSL	BCS
17					
18					
19	Poultry Farm 7	Layer	54 Weeks	WC36	KCS
20					
21					
22	Poultry Farm 8	Layer	63 Weeks	CNC	KCS
23					
24					
25	Poultry Farm 9	Layer	73 Weeks	CNC	KCS
26					
27					
28	Poultry Farm 10	Layer	63 Weeks	CNC	FS
29					
30					
31	Poultry Farm 11	Layer	23 Weeks	CNC	KCS
32					
33					
34	Poultry Farm 12	Layer	28 Weeks	CNC	FS
35					
36					
37	Poultry Farm 13	Broiler	24 Days	CS	FS
38					
39					
40	Poultry Farm 14	Broiler	17 Days	HS	FS
41					
42					
43	Poultry Farm 15	Broiler	21 Days	RS	FS
44					
45					
46	Poultry Farm 16	Broiler	7 Days	RS	FS
47					
48	Poultry Farm 17	Broiler	19 Days	HS	FS
49					
50					
51	Poultry Farm 18	Broiler	35 Days	RS	FS
52					
53					

⁎ Farm Types: KCS = Karachi cage system, FS = Floor system and BCS = Battery cage system.

⁎⁎ Breeds: CNC = Crystal Nick Chick, HS = Hubbard Strain, RS = Ross Strain, CS = Cobb Strain, LSL= Lohmann Selected Leghorn, WC36= Hy-Line W-36 Chicken.

Microbial DNA from the fecal matter was extracted with the QIAamp Fast DNA Stool Mini Kit (Qiagen, Germany), according to the manufacturer's protocol. The final DNA concentrations were determined with Qubit (Thermo Scientific, USA).

### Library preparation and nucleotide sequencing

The V3–V4 region of the bacterial 16S rRNA fragment was amplified by PCR (Klindworth et al., 2013). The nucleotide sequence of the forward primer (341F) in the 5′ to 3′ direction was TCGTCGGCAGCGTCAGATGTGTATAAGAGACAGCCTACGGGNGGCWGCAG, while the reverse primer (785R) sequence was GTCTCGTGGGCTCGGAGATGTGTATAAGAGACAGGACTACHVGGGTATCTAATCC.

The purified amplicons were attached by a second PCR with a dual adapter index with a unique barcode sequence from the Nextera XT Index kit (Illumina Inc., USA) to differentiate each sample. Subsequently, the libraries were quantified to ensure the composition of a sample pool with equimolar amounts of each library. Finally, denaturation of the libraries and PhiX was performed to allow sequencing. The library was quantified and normalized to ensure that the pooled samples contained an equal amount of the library. Pooled samples were denatured with sodium hydroxide, diluted with hybridization buffer, and heated prior to the MiSeq sequencing. Amplicon and control libraries were combined, loaded into a reagent cartridge (Illumina cartridge 2 × 250 MiSeq V2), and subsequently ran using the Illumina MiSeq sequencing platform. The raw sequence data were deposited into the National Center for Biotechnology Information (NCBI) database under BioProject number PRJNA1188055.

### Sequence and data analysis

Sequences obtained from the MiSeq platform were first subjected to quality control analysis. Redundant base pairs were discarded, and truncation length was set to 250 bp using Qiime2. The truncation and denoising steps were done using the Quantitative insights into microbial ecology 2 (QIIME2) plugin DADA2 (2024.5.0) (Callahan et al., 2016) with the Phred score of 30. Set parameters for the quality were ensured by importing the qiime2 artifact and visualizing the quality stats and plots in Q2 plugin demux. The data was subjected to taxonomic assignment using naïve Bayes classifier pre-trained on the SILVA reference database (Emamjomeh et al., 2023) with 99 % operational taxonomic units (OTUs) full-length sequences (v 1.4.2) (Bokulich et al., 2018). Data cleaning and wrangling was done using Python 3.1+ libraries pandas 2.0.3(McKinney, 2010). Phyloseq 1.46.0 was used to calculate the abundance of microbial taxonomic classification (McMurdie and Holmes, 2013). Species evenness and richness were calculated using alpha diversity for all the metrics (Shannon, chao1, observed and Simpson). For the comparison of different metrics in alpha diversity Kruskal-Wallis test and Wilcoxon rank sum test were conducted after adjustments using the Benjamini-Hochberg method. For beta diversity analysis, a wider approach of ordination was adopted and Bray-curtis method was utilized for visualizing non-metric multidimensional scaling (NMDS). For testing various variables Adonis2 was used in vegan 2.6-6.1 R package for calculating permutational multivariate analysis of variance (PERMANOVA) using 999 permutations and subsequently permutational dispersion test (PERMDISP2). For a wider analysis of taxonomic classification heatmaps were plotted using pheatmap 1.0.12. The Spearman method was used to calculate co-occurrence among the population. Apart from this, different R packages i.e., ggplot2 3.5.1(Wickham, 2016), dplyr 1.1.4 (Wickham et al., 2023) and, tidyr 1.3.1 (Wickham et al., 2024) were used to visualize and transform data.

Functional profiling of microbiomes was conducted using Phylogenetic investigation of communities by reconstruction of unobserved states (PICRUSt2) which employed the Greengenes database (Bokulich et al., 2018; McDonald et al., 2024). Annotated pathway data were linked to Kyoto encyclopedia of genes and genomes (KEGG) orthologs and Enzyme commission (EC) numbers to provide functional context. To analyze the pathway abundance differences between broilers and layers and descriptive statistical analysis (i.e.., means, standard deviations, and standard errors for major metabolic processes), statistical comparisons were conducted using the Wilcoxon rank-sum test, with Storey's False discovery rate (FDR) correction applied using the qvalue 2.34.0 package to control for multiple comparisons. Log2 fold changes and percentage differences were calculated to quantify pathway variations, and 95 % confidence intervals were derived using the t-distribution for robust estimation of abundance ranges.

## Results

### Sequencing output

Total 53 samples were analyzed with a total read count of 5,812,925, maximum reads are 3,75,996 and minimum read counts are 5,230 with a median 89,927 reads per sample (Supplementary Table-1).

### Microbial community abundance

**Fig. 2:**
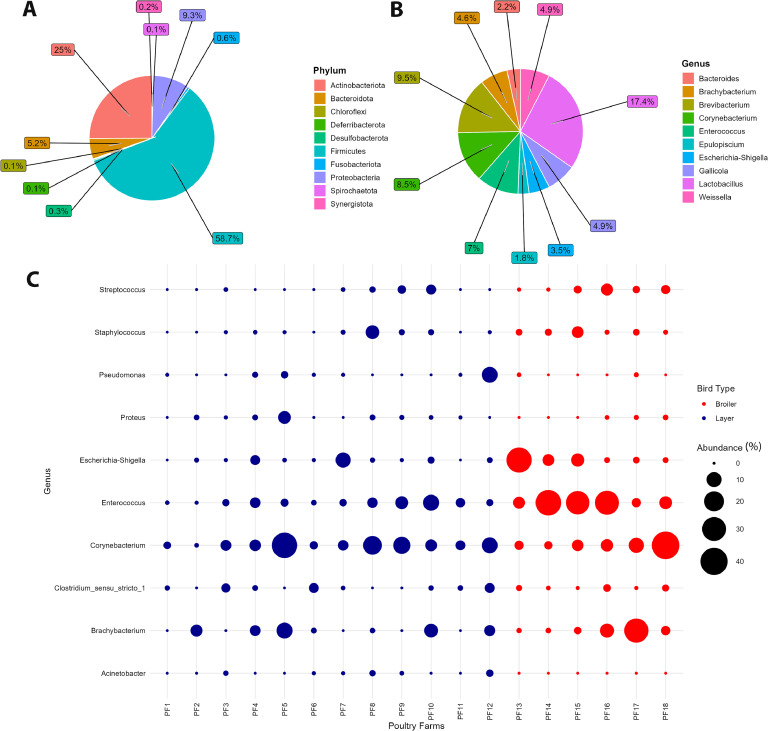
Total abundance in all samples is based on top 10 (A) phyla and (B) genera and (C) total abundance for potentially pathogenic genus in 18 different poultry farms depicted in broilers and layers.

Total abundance was analyzed for bacterial genera and phyla across all samples using Phyloseq. The most dominant phylum, *Firmicutes*, accounted for a substantial proportion of the community, with a total abundance of 156,115 OTUs, representing 58.72 % of the overall microbial population (Fig. 2A). This was followed by *Actinobacteriota*, which contributed 66,552 OTUs to the total abundance, equivalent to 25.03 %, indicating a significant role in the microbial community alongside *Firmicutes. Proteobacteria* ranked third with an abundance of 24,816 OTUs, comprising 9.33 % of the total, followed by *Bacteroidota*, (5.24 %). The remaining phyla contributed relatively smaller proportions, i.e., *Fusobacteriota* (0.59 %), *Desulfobacterota* (0.28 %), *Synergistota* (0.19 %), *Chloroflexi* (0.14 %) and *Spirochaetota* (0.09 %). *Deferribacterota* emerged as the least abundant phylum in the dataset, contributing only 211 OTUs, which corresponds to 0.08 % of the total. *Firmicutes* and *Actinobacteriota* dominated the community, indicating their central role in microbial processes and interactions in poultry. Ten least abundant phyla and genera in all samples were also identified and are listed in Supplementary Table 2 and 3.

The total abundance for community analysis depicted *Lactobacillus* as the predominant genus, accounting for 46,372 OTUs in total abundance, or 17.44 % of the entire community, followed by *Brevibacterium* (9.47 %), *Corynebacterium* (8.50 %), *Enterococcus* (6.95 %), *Gallicola* (4.90 %), *Weissella* (4.88 %), *Brachybacterium* (4.58 %), *Escherichia-Shigella* (3.46 %), *Bacteroides* (2.23 %) and *Epulopiscium* (1.84 %) (Fig. 2B).

The total abundance of potentially pathogenic genera was also analyzed across all poultry farms, as shown in Fig. 2C. Ten different targeted genera were detected at varying abundance levels in the fecal matter. *Enterococcus* and *Corynebacterium* were the most common across all farms, regardless of bird type. *Escherichia-Shigella* was most abundant in broiler farm 13 (PF13), *Staphylococcus* in layer farm 6 (PF6) and broiler farm 15 (PF15), and *Brachybacterium* in broiler farm 17 (PF17).

**Fig. 3:**
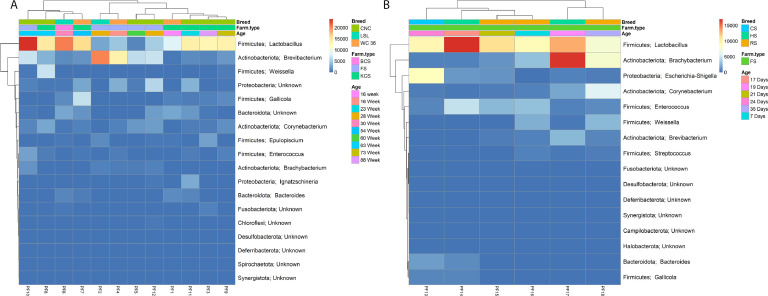
Cluster heatmap of phylum-genera across all the variables of (A) layers and (B) broilers.

Furthermore, the relative abundance for five most abundant phyla and genera were plotted in stacked bar plots depicting their distribution in individual poultry farms separately for layers (Fig. 3A-B) and broilers (Fig. 3C-D). The relative distribution among phyla in both layers and broilers is consistent with total abundance as *Firmicutes* is most frequently distributed in all the poultry farms, followed by *Actinobacteriota* and *Proteobacteria*. The most abundant genus in both groups was *Lactobacillus*, followed by *Enterococcus* in broilers and *Brevibacterium* in layers, whereas *Corynebacterium* was found in both groups.

The OTUs were clustered and annotated through highly abundant genera with all variables (i.e., breed, farm type, and age) depicted in the same plots for layers (Fig. 3A) and broilers (Fig. 3B) to show the relatedness and abundance of taxonomic classifications and interactions as shown in Fig. 3. In layers, *Firmicutes-Lactobacillus* were highly abundant in all the KCS farm types and one (only) BCS farm type, irrespective of the breeds. Overall, in FS farm type, *Actinobacteriota-Brevibacterium* was moderately abundant in all farms except one farm (PF-10) where *Firmicutes-Lactobacillus* was most abundant. This detailed distribution clearly reflects the varying microbial composition in layers, with specific taxa being more abundant depending on the variables (Fig. 3A).

In broilers, all the poultry farms utilized the same FS farm type (Fig. 3B). Similar to layers, *Firmicutes-Lactobacillus* generally exhibited high abundance and being most prevalent in 17-day-old birds from the HS breed followed by 19-day-old with the same breed and 21-day-old RS breed. *Firmicutes-Lactobacillus* also shares the highest prevalence place with *Escherichia-Shigella* in 24-day-old birds of CS breed and with *Actinobacteriota-Brachybacterium* in 35-day-old birds of RS breed. Interestingly, the fecal matter of the 19-day-old HS breed had the *Actinobacteriota-Brachybacterium* as the most abundant bacterium, followed by *Firmicutes-Lactobacillus*.

### Alpha diversity

**Fig. 4:**
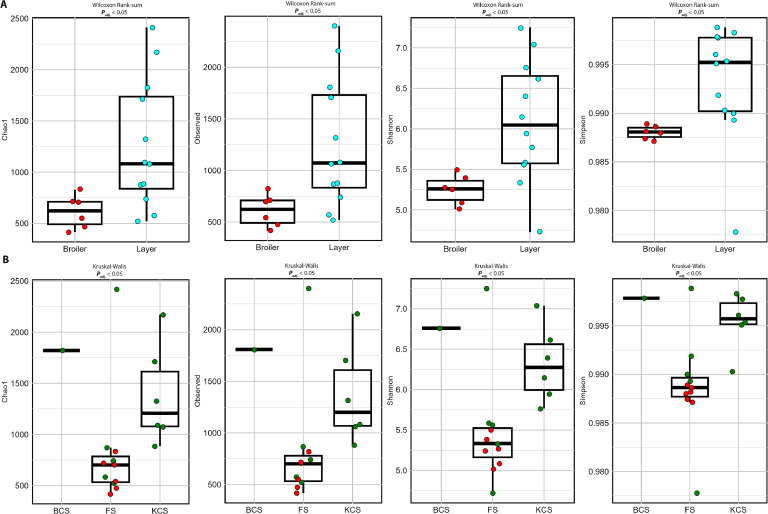
Box plot representing alpha diversity metrics calculated for richness (Observed and Chao1) and evenness (Shannon and Simpson) for (A) bird types, (B) farm types. Non-parametric Kruskal-Wallis test and Wilcoxon rank sum test using Benjamini-Hochberg method for adjusted p-values were calculated. Each round spot depicts each Poultry farm in the group according to diversity distribution. In panel B, layers are represented by green dots and broilers by red dots.

Alpha diversity metrics including Observed, Chao1, Shannon and Simpson, were calculated for two groups of layers and broilers and three farm types. The species richness, evenness and distribution in population were also measured by running non-parametric Kruskal-Wallis test and Wilcoxon-rank sum test as independent variables after running Benjamini-Hochberg correction for adjusted p-values and control of FDR in multiple comparisons. The results from both tests indicated a significant difference across all matrices in both groups. Layers consistently possessed higher richness (Observed and Chao1), more diverse microbial community and a greater dominance of species (Shannon and Simpson) than broilers indicated by increased median values in the boxplots as shown in Fig. 4A.

The same four alpha diversity metrics (Fig. 4B) were also calculated for different farm types (i.e., FS, KCS and BCS) to establish the influence of this variable on the fecal microbiota. KCS farm type consistently possessed higher richness (Observed and Chao1), more diverse microbial community, and greater dominance of species (Shannon and Simpson) than FS and BCS as shown by higher medians as shown in Fig. 4B.

### Beta diversity

**Fig. 5:**
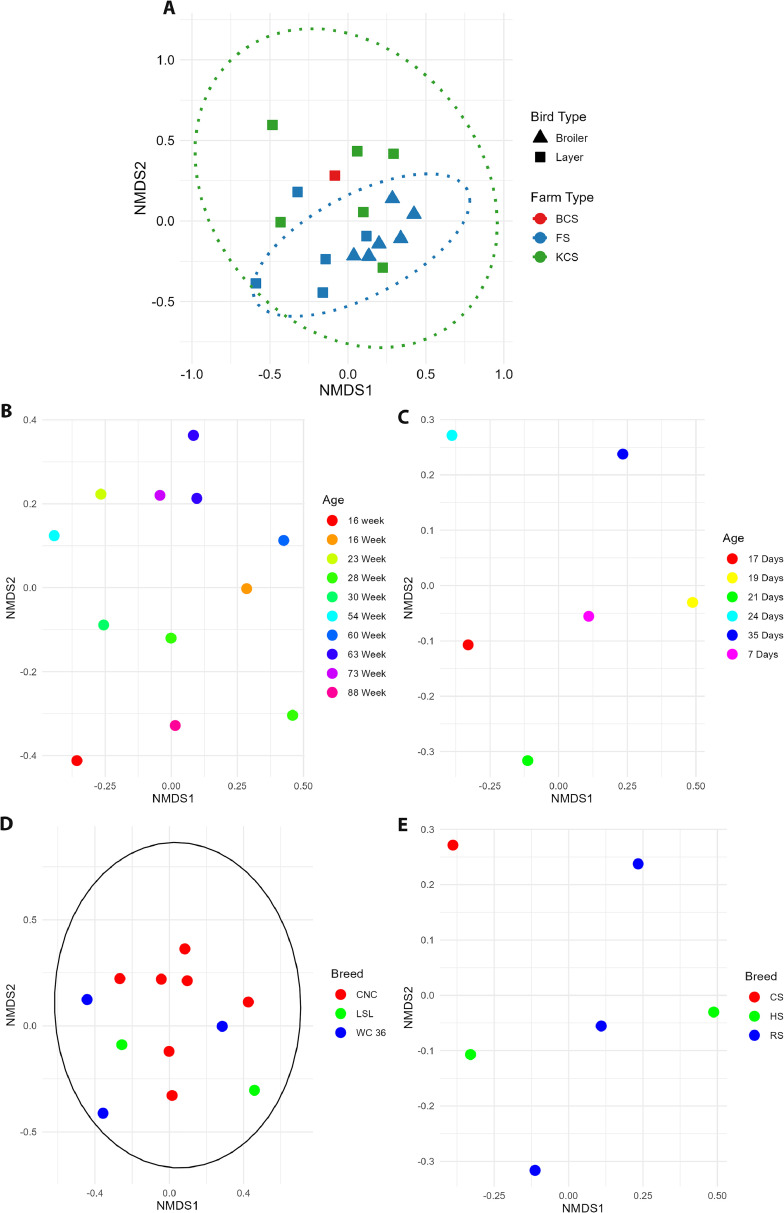
Beta diversity metrics. (A) NMDS ordination conducted on the basis of Bray-Curtis dissimilarity in bird type (layers vs broilers), with distinct separation between bird types (PERMANOVA, *p* = 0.005) and farm type (FS, BCS, KCS) (*p* = 0.039). Ellipses in the plot represents 95 % confidence intervals for each farm type, illustrating the spread of microbial community composition within each farm. NMDS ordination for microbial composition in the community based on age for (B) layers and (C) broilers, breeds in (D) layers and (E) broilers.

To explain the microbial diversity between layers and broilers, other parameters were tested and cross verified to determine the most influential and dependent variable effecting the diversity. A combined and then individual analysis indicated coherent results as shown in Fig. 5. In a combined approach PERMANOVA test using the Bray-Curtis dissimilarity matrix was run to assess the impact of farm type, bird type and age on microbial community structure. Bird type explained 10.08 % of the variation which is statistically significant (*p* = 0.002) (Fig. 5A). The F-statistic value of 1.8951 showed a meaningful difference in each bird type. With a variation of 5.65 % in the microbial community and non-significant results (*p* = 0.360), age was not a factor influencing the microbial diversity. Farm type accounted for 15.14 % of the variation in microbial community structure, yielding a statistically significant finding (*p* = 0.007). The F-statistic of 1.4231 indicates that the diversity among various farm types exceeds the variability within each farm type (Fig. 5A). Additionally, two PERMANOVA tests were also conducted both including bird type and excluding bird type based on the farm type which also showed significant differences influenced by both bird type and farm type. Although farm type and bird type are responsible for the statistically significant variation, the residuals signify the unexplained variation, constituting 69.13 % of the total variation due to other unknown or unmeasured factors.

In layers, age (Fig. 5B) and breed (Fig. 5D) accounted for 82.89 % (*p* = 0.177) and 17.46 % (*p* = 0.633) of the variance in microbial communities, respectively, but this was not statistically significant (Fig. 5B). In broilers, the age (Fig. 5C) and breed (Fig. 5E) contributed to merely 5.65 % (*p* = 0.313) and 17.46 % (*p* = 0.633) of the difference in microbial composition, a finding that was also not statistically significant. To analyze the effect of age or breeds in depth, homogeneity of multivariate dispersions (PERMDISP2) with Bonferroni corrections was checked across breeds and age in layers and broilers separately. The PERMDISP2 analysis for age in broilers yielded inconclusive results with no values for the F-statistic and associated p-values, indicating no detectable within-group variation across age groups. This suggested a lack of variability between age groups in broilers, aligning with the earlier PERMANOVA results where age was not a significant factor in explaining microbial community differences. For age group in layers, the PERMDISP2 test yielded an F-value of 1.066e +30 and a p-value of 0.115 (non-significant). Although approaching marginal significance, the results indicate that age groups in layers do not exhibit significant differences in dispersions, suggesting a limited effect of aging on community composition. The PERMDISP2 analysis for breeds in broilers revealed significant differences in dispersion (*F* = 8.3825, *p* = 0.001389), indicating a community variability that is markedly greater among breeds in broilers. Breeds in layers were also found to affect community dispersion significantly (*F* = 6.0326, *p* = 0.028). This indicates that the microbial communities linked to various breeds in broilers and layers display unique spatial distributions, affirming that breed significantly influences microbial diversity.

### Correlation

**Fig. 6:**
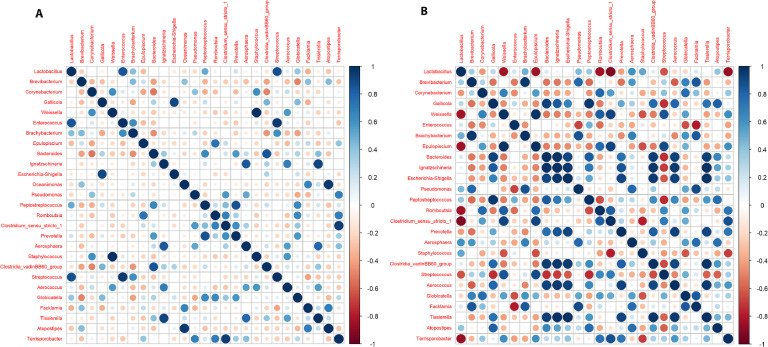
Correlation plot between genera in (A) layers and (B) broilers calculated by Pearson correlation matrix. The intensity of color transition depicts the more positive (blue) and negative (red) relationships between genera across both groups.

The correlation analysis of microbial genera in poultry, based on Pearson's correlation coefficient, was used to present the distinct microbial interactions in layers and broilers (Fig. 6). Correlation matrices were computed by aggregating OTU data at the genus level and normalizing the counts using DESeq2 to adjust for sequencing depth. In layers, strong positive correlations (i.e., *r* > 0.8) were exhibited by 11 genus combinations (Fig. 6A). Some of the more prominent include *Lactobacillus* and *Enterococcus* (*r* = 0.85), *Lactobacillus* and *Streptococcus* (*r* = 0.87), *Gallicola* and *Escherichia-Shigella* (*r* = 0.91), *Weissella* and *Staphylococcus* (*r* = 0.997), *Enterococcus* and *Streptococcus* (*r* = 0.96), *Oceanimonas* and *Atopostipes* (*r* = 0.96), *Clostridium_sensu_stricto_1* and *Terrisporobacter* (*r* = 0.94). However, no strong negative correlations (i.e., *r* < −0.8) were found in the layers’ fecal microbiome.

In broilers, the microbiota exhibited more competitive dynamics, with the strongest negative correlations (i.e., *r* < −0.8) being observed for *Lactobacillus*, including *Lactobacillus* and *Epulopiscium* (*r* = −0.84), *Lactobacillus* and *Weissella* (*r* = −0.83), *Lactobacillus* and *Romboutsia* (*r* = −0.84), *Lactobacillus* and *Clostridium sensu stricto 1* (*r* = −0.92), and *Lactobacillus* and *Terrisporobacter* (*r* = −0.86). However, strong positive correlations were identified among multiple genera, with 39 combinations having an r-value greater than 0.8 (Fig. 6B). Notably, *Gallicola* with *Faklamia* (*r* = 0.85), *Bacteroides* (*r* = 0.93), *Ignatzschineria* (*r* = 0.84), *Peptostreptococcus* (*r* = 0.97), and *Atopostipes* (*r* = 0.8), *Weisella* with *Epulopiscium* (*r* = 0.99), *Romboutsia* (*r* = 0.83), *Clostridium_sensu_stricto_1* (*r* = 0.92) and *Streptococcus* (*r* = 0.86), and *Escherichia-Shigella* with *Bacteroides* (*r* = 0.90), *Ignatzschineria* (*r* = 0.97), *Prevotella* (*r* = 0.91), *Clostridia_vadinBB60_group* (*r* = 0.96), *Aerococcus* (*r* = 0.92) and *Tissierella* (*r* = 0.99).

### Co-occurrence network analysis

**Fig. 7:**
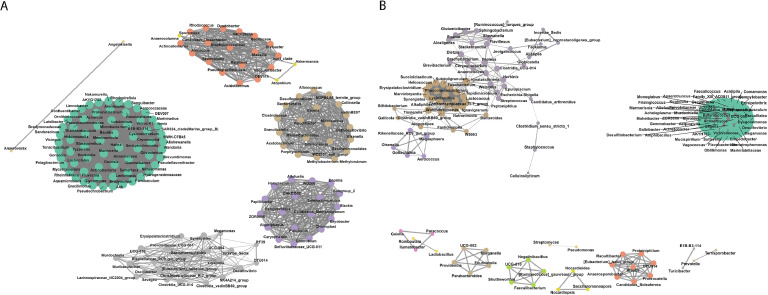
Co-occurrence network graphs representing microbial top 150 genera in (A) layers, and (B) broilers. Nodes represent microbial genera, and edges represent significant co-occurrence relationships (Spearman correlation, *r* > 0.8 or *r* < −0.8, *p* < 0.05). Node size corresponds to relative abundance, and edge thickness reflects the strength of correlation. Nodes are color-coded by different clusters and edges are represented by lines among nodes. Clusters represent groups of co-occurring genera, emphasizing potential functional or ecological associations.

The co-occurrence network was constructed using OTUs which represented the relationship between bacterial genera of layers (Fig. 7A) and broilers (Fig. 7B). The co-occurrence relationship was determined by Spearman correlation using a monotonic function and cutoff value set to 0.8 to show strong positive relationships and reduce noise. The co-occurrence network analysis of bacterial taxa within layer poultry microbiomes revealed a highly interconnected microbial community, underscoring complex interspecies interactions. Prominent genera such as *Actinobacteria, Aerococcaceae*, and *Anaerostipes* exhibited significant positive co-occurrence with multiple taxa, suggesting their central role in maintaining microbial network stability. Additionally, *A4b, Alishewanella*, and *Bradymonadaceae* formed distinct clusters linked through shared ecological niches, indicating potential functional dependencies or synergistic relationships. Negative co-occurrence was observed between *Ralstonia* and *Bacteroidales*, highlighting potential competitive dynamics within the gut environment. The co-occurrence network analysis of the broiler gut microbiome revealed significant interactions among bacterial genera, highlighting both core members and potential pathogenic associations. Highly connected genera such as *Acholeplasma, Acinetobacter*, and *Anaerotruncus* emerged as central nodes in the network, indicating their pivotal ecological roles. Notably, *Escherichia-Shigella, Campylobacter*, and *Staphylococcus*, which include potential pathogens, formed associations with commensal genera such as *Lactococcus, Bacteroides*, and *Clostridium-sensu-stricto-1*, suggesting microbial interactions that may influence gut health and pathogen colonization.

### Predicted functions

**Fig. 8:**
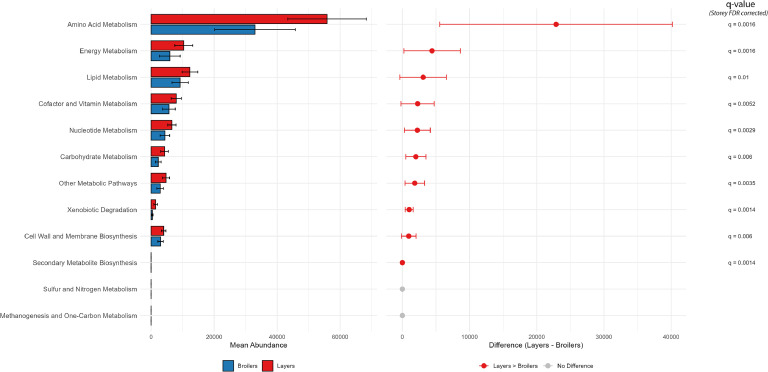
Mean abundances of major metabolic pathways predicted by PICRUSt analysis in layers and broilers. The pathways are arranged in descending order of mean abundance. Each bar represents the mean abundance of a pathway for layers (red) and broilers (blue), with error bars indicating 95 % confidence intervals for the mean. Above the bars, the adjusted q-values (FDR corrected) are annotated for pathways where *q* < 0.05. In the adjacent panel, a dot plot visualizes the difference in mean abundance (layers - broilers) for each pathway, with error bars showing the 95 % confidence intervals for the differences. Pathways significantly enriched in layers (*q* < 0.05) are indicated by red dots, and q-values are annotated to the right of the error bars.

To assess the variability in metabolic pathways between layers and broilers, PICRUSt2 analysis was conducted separately for each bird type. In total, 2,913 EC pathways were identified in layers, while 2,804 were identified in broilers. Additionally, 10,544 KEGG orthologs were detected in layers, compared to 9,950 in broilers. Metabolic pathway enrichment analysis using MetaCyc identified 489 pathways in layers and 485 pathways in broilers, which were subsequently classified and categorized into 12 primary metabolic processes (Fig. 8). The analysis revealed significant differences in metabolic pathway abundances between layers and broilers across multiple processes. Amino acid metabolism showed a mean abundance of 55,837 in layers compared to 32,960 in broilers. Carbohydrate metabolism was also higher in layers (4,266) than in broilers (2,254). Similarly, cell wall and membrane biosynthesis demonstrated a marked increase in layers, reaching 5,902 compared to 3,015 in broilers. Cofactor and vitamin metabolism was more abundant in layers (7,950) than in broilers (5,680). Energy metabolism displayed a mean abundance of 10,348 in layers versus 5,926 in broilers. Lipid metabolism was also elevated in layers, with an average of 12,300 compared to 9,207 in broilers. Nucleotide metabolism presented a mean abundance of 6,580 in layers versus 4,351 in broilers. Additional metabolic pathways were higher in layers (4,721) than in broilers (2,873). Secondary metabolite biosynthesis showed a significant increase in layers, with a mean abundance of 2.32 compared to 0.06 in broilers. Lastly, xenobiotic degradation had a mean abundance of 1,428 in layers and 409 in broilers. Overall, these findings highlight significantly higher abundances across these metabolic pathways in layers, indicating distinct metabolic profiles between layers and broilers. The analysis also identified four metabolic pathways unique to layers: Long chain fatty acid biosynthesis I, Superpathway of seleno-compound metabolism, Sciadonate biosynthesis, and Superpathway of neomycin biosynthesis.

## Discussion

This study provided a comprehensive description of the microbiome in the fecal matter of 6 broiler farms and 12 layer farms in an attempt to provide a baseline picture of the microbial taxonomical diversity in various breeds, age, and farm types in commercial production at the particular region. Now the most common method to study bacterial diversity and composition is the 16S ribosomal RNA or rRNA gene amplicon based next generation sequencing (NGS) using the Illumina platform (Gupta et al., 2019). Analyses of gut microbiota have generally been performed by sampling the gut contents of birds, requiring the sacrifice of animals. However, in this study, we used a noninvasive method, as we sampled only feces, and it was unnecessary to sacrifice any bird. According to Stanley et al. (2015) (Stanley et al., 2015), there is a high correlation between the fecal microbiota and the cecal microbiota of birds. During the last decade, advancements in understanding of the microbiome of poultry gut have been significant, however the degree of variation in different studies remain high (Stanley et al., 2013). This is due to difference in parameters and influencing factors of both the host and the environment, including location, age, gut region, sex, diet, farm management system, breed and so on (Kers et al., 2018). Identifying these shared taxa and elucidating their function within birds could lead to a greater understanding of the microbial ecology of the chicken gut and the development of technologies that could be widely applied to improve chicken health or productivity (Glendinning et al., 2019).

In the present study, the most dominant phylum found was *Firmicutes*, followed by *Actinobacteriota* and *Proteobacteria* which is in line with the previous studies where dominance of similar phyla were observed in different part of the gut such as the cecal microbiomes of both broiler and layer (Mancabelli et al., 2016; Ocejo et al., 2019; Qi et al., 2019) as well as ileal feces (Alvarenga et al., 2023). Like our results, irrespective of bird types a similar pattern of the dominant phylum (*Firmicutes, Bacteroidetes, Actinobacteria*, and *Proteobacteria*) was reported in the previous studies of chicken (Pan and Yu, 2014; Ranjitkar et al., 2016; Willson et al., 2022) as well as turkey (Wilkinson et al., 2017). The most abundant genera, in no particular order, in the broiler were *Brachybacterium, Corynebacterium, Enterococcus, Escherichia-Shigella* and *Lactobacillus*, while in the layers were *Brevibacterium, Corynebacterium, Gallicola, Lactobacillus* and *Weissella*. Other studies found that the most abundant genera in all groups (layers and broilers) were, *Lactobacillus* and *Escherichia/Shigella* in fecal matter (Alvarenga et al., 2023), *Ruminococcus*, unclassified *Clostridiales, Faecalibacterium, Escherichia* and *Bacteroides* in the cecum (Willson et al., 2022), *Lactobacillus, Escherichia/Shigella, Bacteroides* and *Faecalibacterium* in ileal feces (Alvarenga et al., 2023). In turkey, gut sequencing data showed an abundance of *Lactobacillus, Streptococcus*, and *Clostridium* XI irrespective of location and age (Wilkinson et al., 2017). Simply due to their dominance, taxonomic groups of high abundance may have a greater impact on nutrient assimilation of the host and host physiology than single low-abundance phylotypes (Apajalahti and Vienola, 2016).

In the current study, *Lactobacillus* was the most prevalent genus in both layers and broilers, regardless of variables, consistent with previous studies where it had also been reported as the most abundant (Zhao et al., 2013)**.** *Lactobacillaceae* (belonging mainly to the genus *Lactobacillus*) represented most of the *Firmicutes* at all ages and in all segments of the gut except the cecum (Ranjitkar et al., 2016). *Lactobacillus* could affect lipid metabolites which impact intestinal permeability and activate the intestine-brain-liver neural axis to regulate glucose homeostasis (Qi et al., 2019). Interestingly, antibiotic treatment has been shown to alter gut and fecal bacterial species composition in chicken towards an increased abundance of *Lactobacillus* spp. *Clostridiales* and *Enterobacteriaceae* (Mancabelli et al., 2016). In Pakistan, poultry farming practices routinely include supplementing feed with antimicrobials as growth promoters (Ur Rahman and Mohsin, 2019; Umair et al., 2022). The type of antibiotic used can significantly influence the microbial composition. However, since the study focuses on commercial poultry farms, detailed data on antibiotic regimens, including type, dosage, and administration, were not available. Moreover, a large percentage of the on-farm antimicrobial usage is of critical importance because the used antimicrobial classes have high to highest priority for human medicine (Umair et al., 2021). Most intestinal microbes do not directly affect the host, but their metabolites do. The metabolites of the intestinal microbiota include various kinds of vitamins, amino acids, short-chain fatty acids, and various gases (carbon dioxide, ammonia, methylene, ammonia, indole), and they also maintain the pH of the intestinal environment (Qi et al., 2019).

Host genotype and gender are among the factors that influence the composition of the gut microbiota (Zhao et al., 2013). In the present study, age was not one of the significant influencing factors, this can be explained by the fact that this was not an experimental study where the microbiomes of different age groups, under a controlled diet and husbandry were analyzed. Whereas, breeds, irrespective of the bird type, significantly impacted microbial communities displaying unique distributions. The genetic background of the host has been recognized as a factor that might influence intestinal microbiota composition. Commercial selection programs for high production may result in the co-microevolution of the microbiota and immune system of the host (Yang et al., 2017), although other factors, such as differences in exposure to microbial communities, cannot be excluded (Kers et al., 2018). Farm type accounted for 15.14 % of the variation in microbial community structure, yielding a statistically significant finding. KCS differs from BCS not only in the manual collection of eggs and waste but also with the bird population in each cage but which is similar in restricting the bird mobility. In contrast, FS allowed the birds to exhibit natural behaviors of perching and dust bathing. Housing or farm type of production system have been reported to influence microbiota composition (Kers et al., 2018).

This study from Pakistan—where poultry is a vital part of the food and agricultural economy—provides valuable initial insights into the microbiota of commercial poultry farms. While the findings lay a strong foundation for future research, several aspects should be expanded upon in subsequent studies. Fresh fecal samples were collected with care to avoid litter contamination, though minor mixing (particularly in FS-housing type), cannot be entirely excluded. A broader geographical range, including more farms from various regions, as well as factors such as feed source and composition, medication, and environmental conditions, should be considered in future work. Additionally, the current study focused on the floor system for broilers and included only one battery cage system for layers, so investigating a wider variety of farm types and environments would enrich the data. More samples will also be needed to strengthen conclusions regarding the effects of age, especially in relation to food supplementation and antibiotic treatments. Despite these limitations, this study sets an important precedent for microbiome research in the Pakistan's poultry sector and highlights consistent taxa within the chicken gut, paving the way for future advancements with potential applications in improving poultry health and productivity through microbiome-targeted strategies.

## Declaration of competing interest

The authors declare that the research was conducted in the absence of any commercial or financial relationships that could be construed as a potential conflict of interest

## Appendix Supplementary materials

Image, application 1

Image, application 2

## Footnotes

Supplementary material associated with this article can be found, in the online version, at doi:10.1016/j.psj.2025.105089.
