# Supplementary material for: Fecal microbiota landscape of commercial poultry farms in Faisalabad, Pakistan: A 16S rRNA gene-based metagenomics study
Source: Poult Sci. 2025 Mar 23;104(6):105089. doi: 10.1016/j.psj.2025.105089 (PMC12002918; doi:10.1016/j.psj.2025.105089)

**Karachi Cage System (KCS):** Farmers often rely on sophisticated layered cage systems, ranging from smaller setups that hold 96 birds per cage to larger operations that can accommodate up to 100,000 birds. The height of one tier is typically around 45 to 60 cm. These systems often have 3 to 5 tiers, depending on the size of the poultry house. Each individual cage consists of 12 compartments, and within each compartment, there are 8 chickens, resulting in a total of 96 birds per cage. This setup not only conserves space but also ensures that each bird has its designated area, reducing overcrowding and stress, which are critical for maintaining high egg yield.

The manure produced by the chickens accumulates on the ground beneath the cages and is manually removed, ensuring proper sanitation and minimizing the risk of diseases. The feeding system is manually adjusted to ensure that all chickens receive the appropriate amount of feed, and the water lines are similarly controlled to provide adequate hydration. Egg collection is also done by manually and any weak or underperforming birds are manually removed from the system, allowing farmers to maintain a healthy flock and enhance overall productivity. Despite the manual labor involved, these systems are highly effective for large-scale poultry farming, offering a balance between automation and hands-on management.


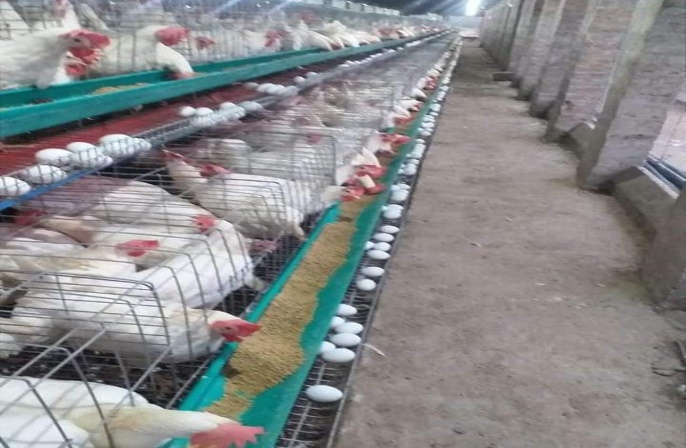

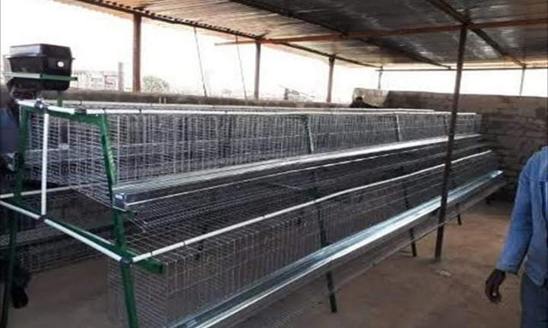

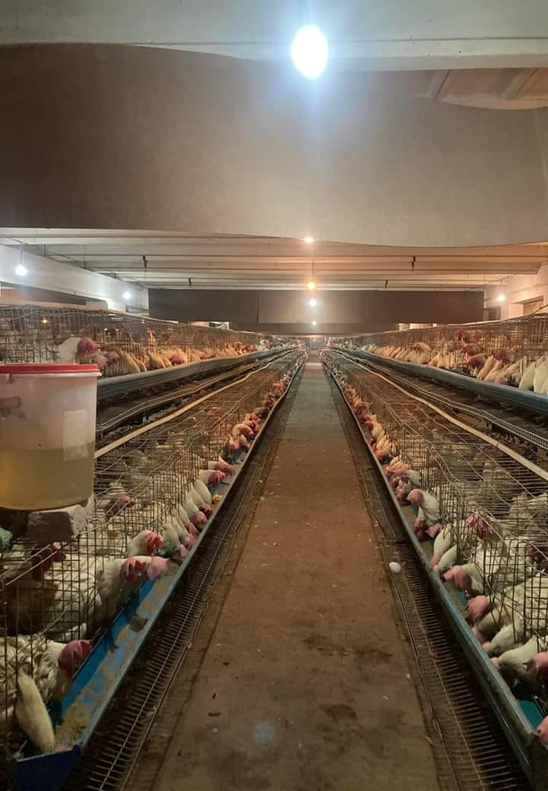


**Battery Cage System (BCS):** It is a popular housing system in commercial poultry farming, particularly in large-scale egg production. It is designed to maximize space efficiency, ease of management and productivity. Modern battery cages are typically designed to hold 8 to 15 hens per compartment, with each bird receiving around 430 to 600 square centimeters of space. In larger battery cage systems (stacked in 3 to 8 tiers), the height of each cage can exceed 75 cm, especially in multi-tiered setups where airflow and waste management are key considerations. This vertical arrangement not only optimizes space but also enhances the overall efficiency of farm management, allowing for higher stocking densities and easier access to the birds.

Feeding and watering systems are fully automated, ensuring a consistent supply of food and water to the birds. Additionally, the cages are equipped with automated manure removal systems, which help to maintain cleanliness and reduce the risk of diseases. The collection of eggs is also automated, streamlining the egg production process and reducing labor costs. Furthermore, many modern battery cage systems include climate control features, such as automatic temperature regulation, which helps to maintain optimal conditions within the poultry shed. It is sometimes criticized because of the limited space and movement available to the hens that often results in concerns about the birds' ability to express natural behaviors, such as dust bathing, perching, and nesting.


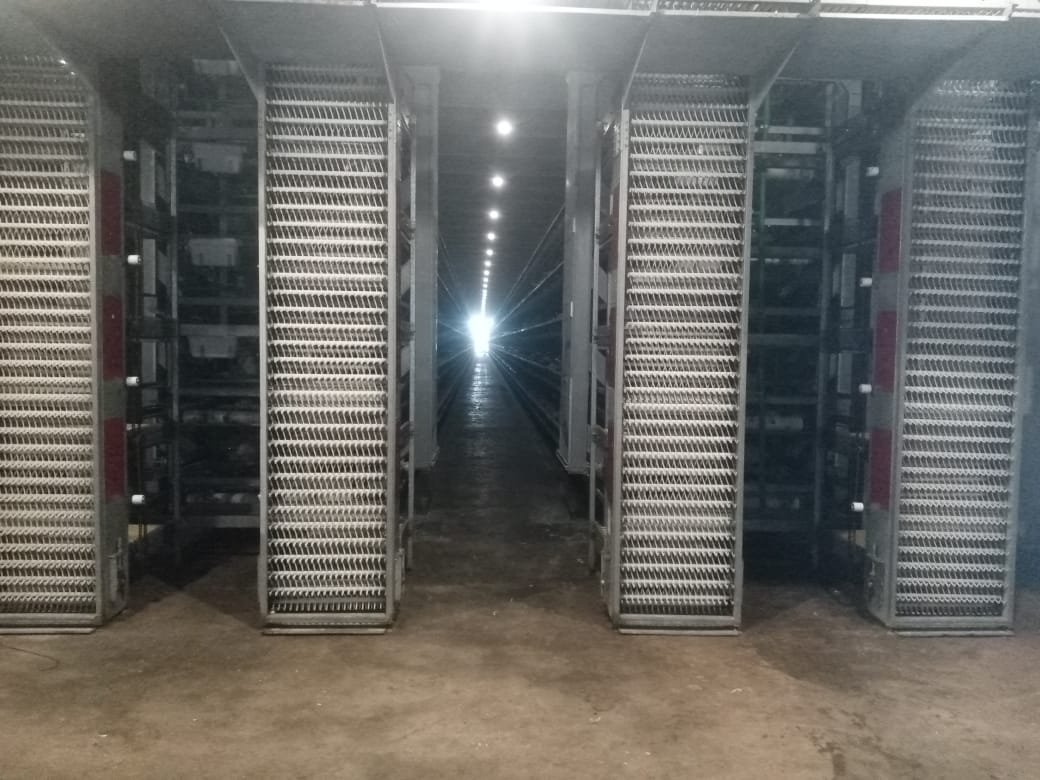

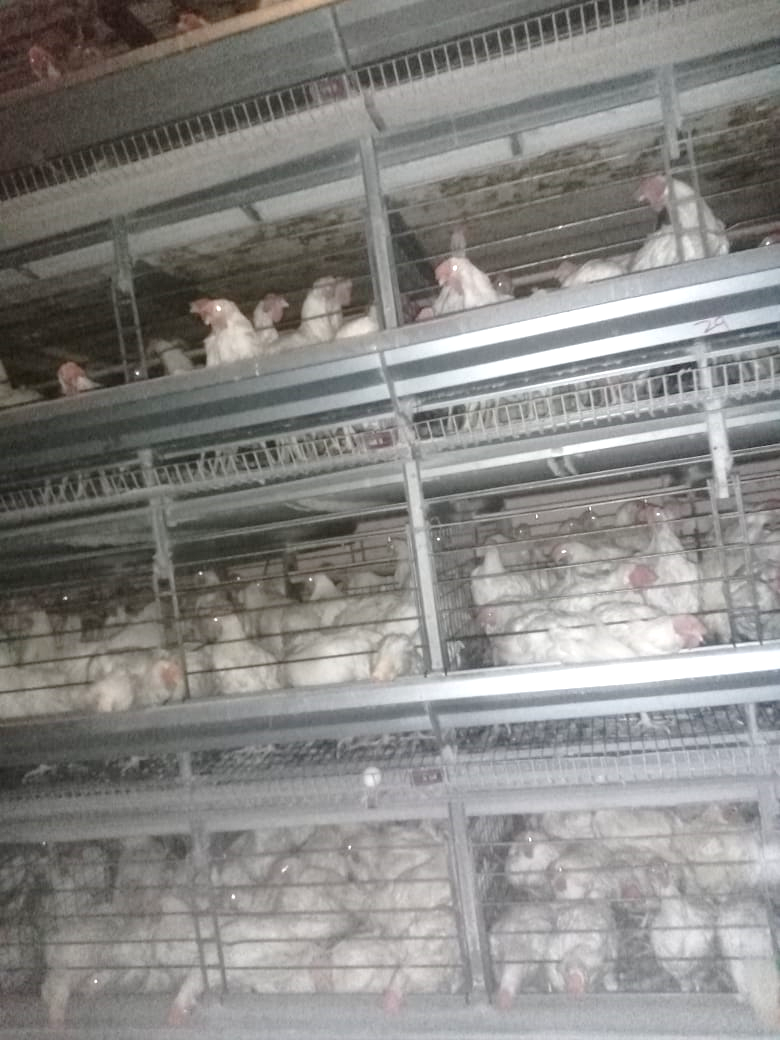

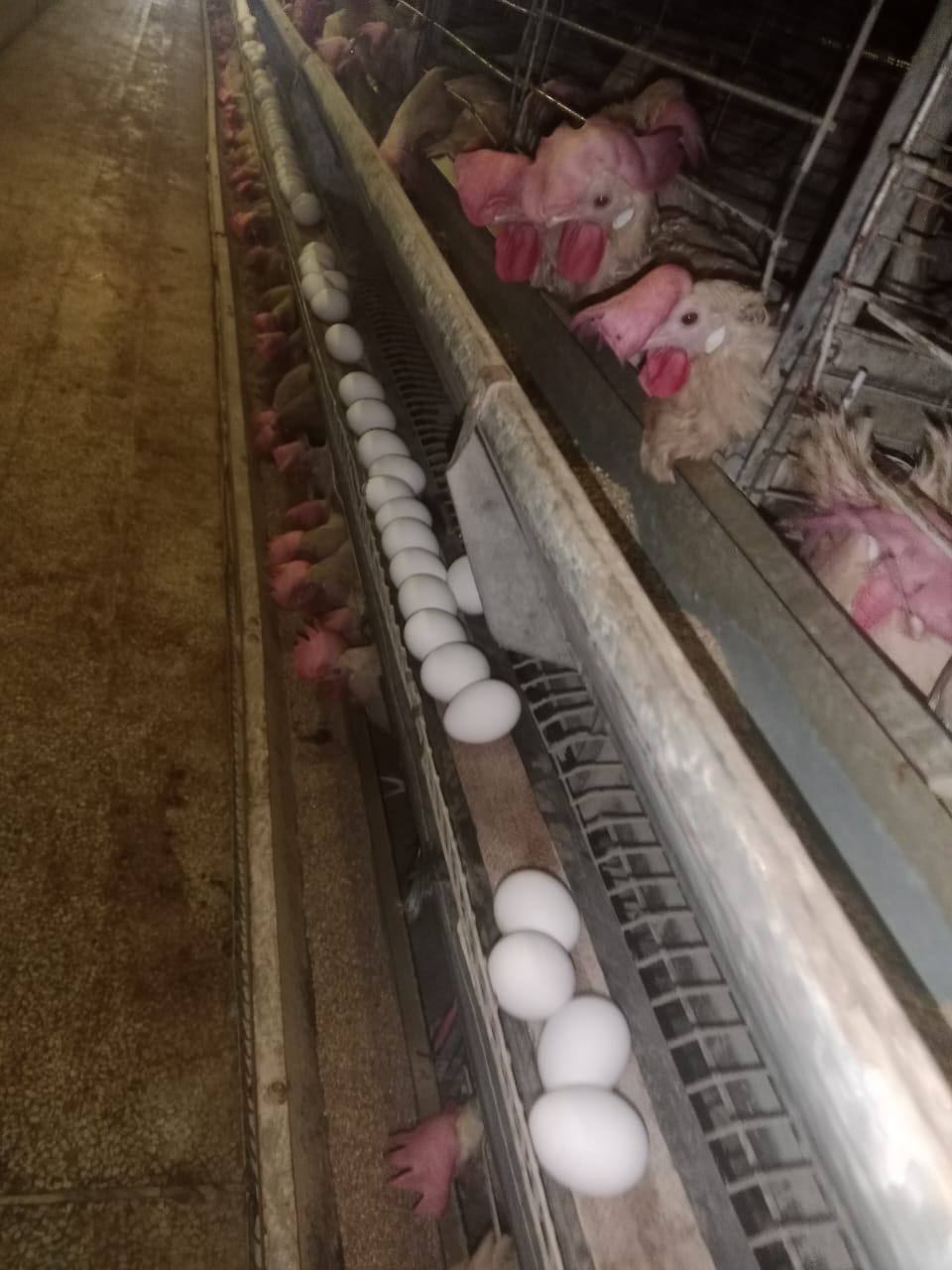


**Layer Floor System:** In a floor system for layer chickens, the birds are raised on the floor rather than in cages, allowing them to engage in more natural behaviors such as perching and dust bathing. The bedding material, typically wood shavings, straw, or rice hulls, plays a critical role in absorbing moisture and controlling odors, while also preventing foot lesions and maintaining hygiene. Feeding and watering are often managed manually, with feed being distributed through troughs or hanging feeders and water provided via bell drinkers or nipple drinkers to ensure the hens have constant access to nutrition and hydration.

Nesting areas, usually elevated to prevent litter contamination, are strategically placed throughout the poultry house and lined with soft materials like straw to provide a comfortable space for hens to lay their eggs. Egg collection is generally done manually, with workers gathering eggs regularly to prevent breakage and maintain quality. Temperature regulation is managed through ventilation systems, fans, heaters, and cooling pads to keep the environment comfortable, with an ideal temperature range of around 18-22°C to support optimal health and egg production. This integrated system ensures the welfare of the hens while maximizing productivity.


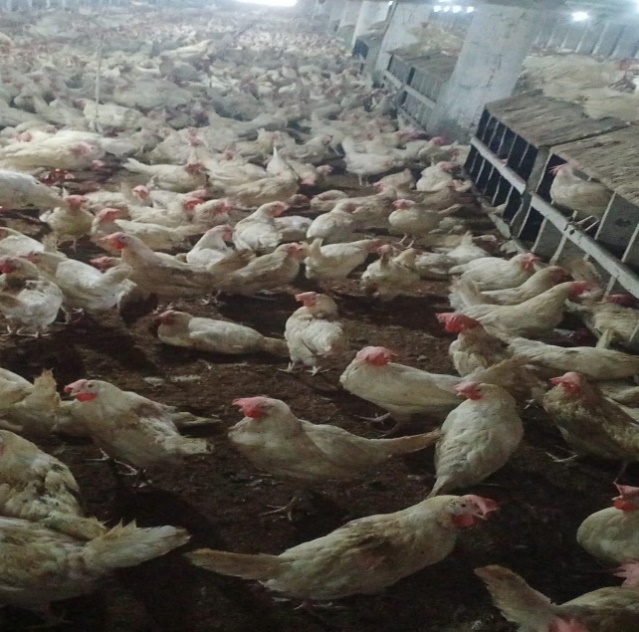

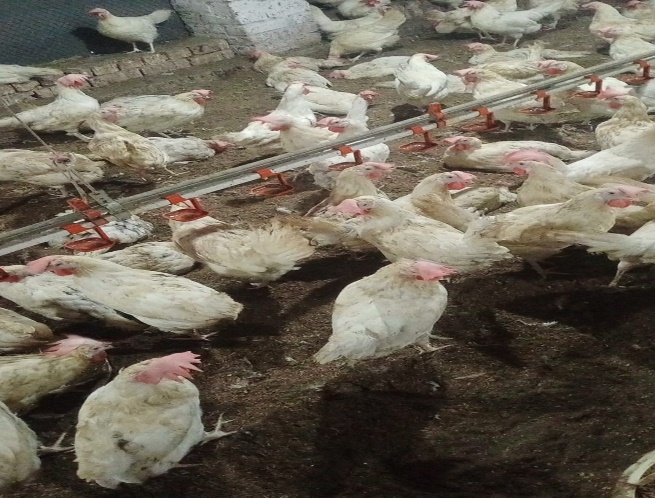

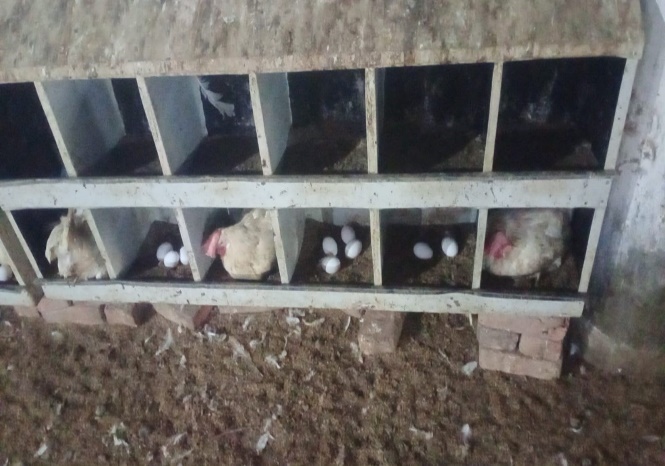


**Broiler Floor System:** In a broiler housing environment, several key systems work together to ensure optimal conditions for the health and growth of broiler chickens. One important aspect is the use of bedding material, which plays a crucial role in providing comfort, absorbing moisture, and maintaining hygiene. Common bedding materials include wood shavings, rice hulls, chopped straw, and peat moss, each offering varying levels of absorbency and insulation. The litter must be managed carefully to prevent wet spots and ammonia buildup, which can lead to respiratory issues and footpad lesions in the birds. In the feeding system, where automated pan feeders are widely used for even feed distribution, allowing easy access for the broilers at different stages of growth. The watering system is also automated, with nipple drinkers being the most common type, minimizing water spillage and keeping the litter dry.

Temperature regulation is equally critical, especially during the early brooding stage when chicks require a higher temperature (32-34°C). As the birds grow, the temperature is gradually reduced, with ideal levels being around 18-22°C. Modern broiler houses use automated systems to monitor and control temperature, employing ventilation systems for airflow, heating systems like gas brooders for warmth, and cooling systems such as evaporative pads and misting devices in hotter climates.


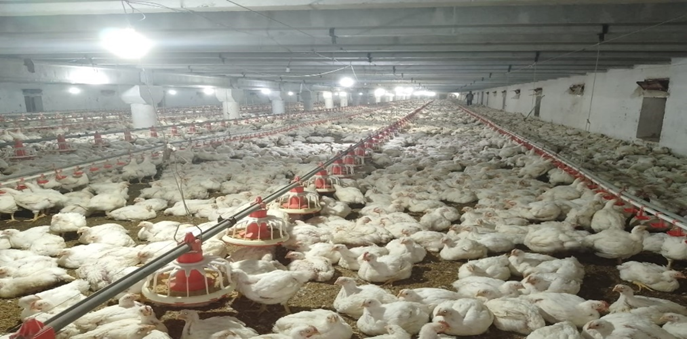

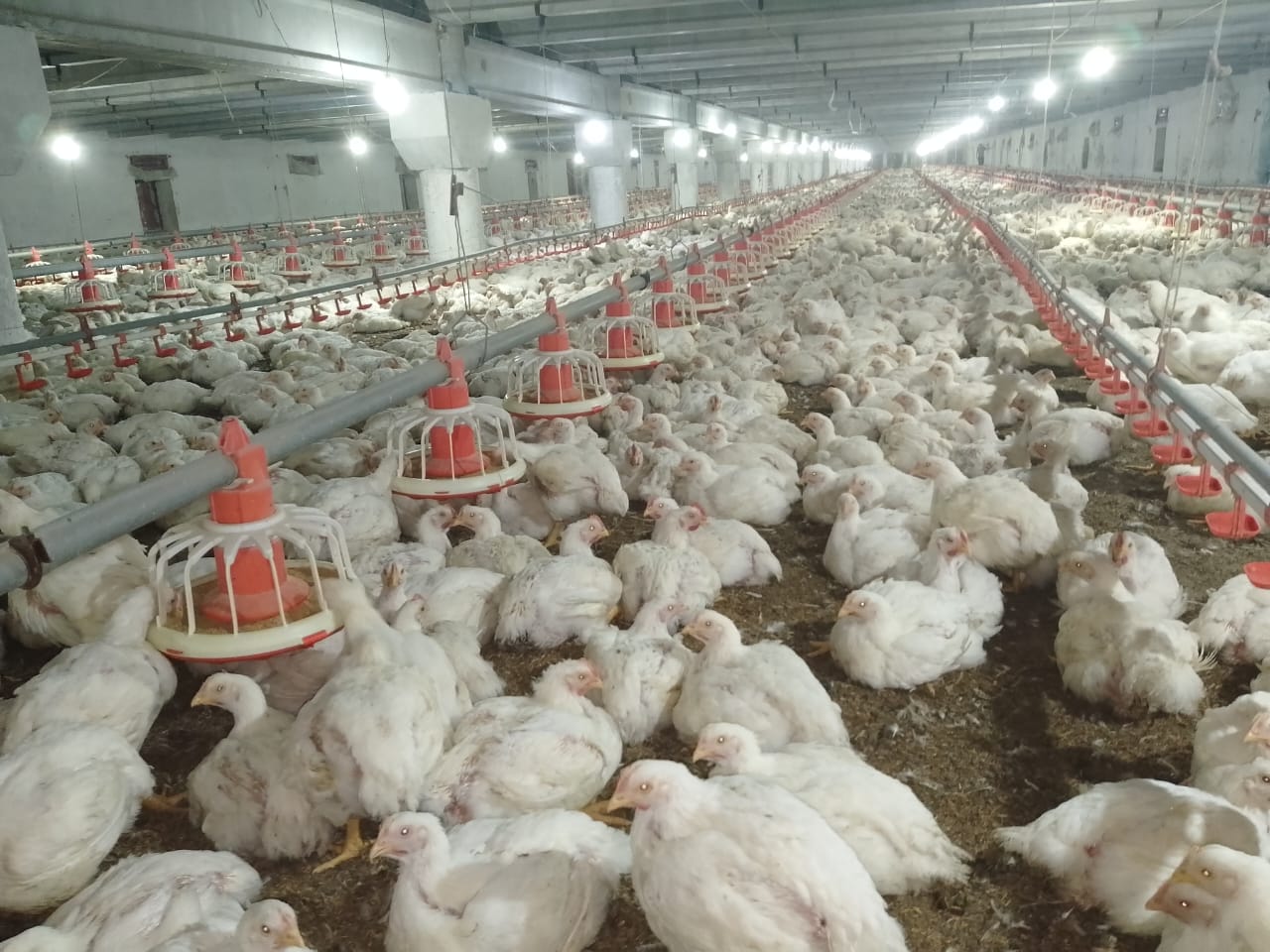

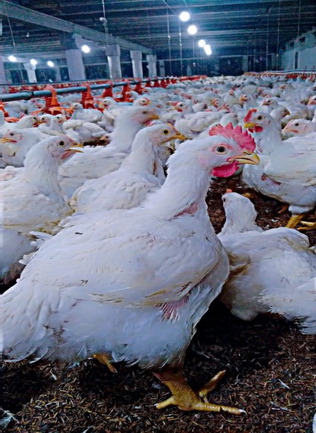

Supplement: Supplementary file 1 [file mmc1.docx]
