# Supplementary material for: Fecal microbiota landscape of commercial poultry farms in Faisalabad, Pakistan: A 16S rRNA gene-based metagenomics study
Source: Poult Sci. 2025 Mar 23;104(6):105089. doi: 10.1016/j.psj.2025.105089 (PMC12002918; doi:10.1016/j.psj.2025.105089)
Supplement: Supplementary file 2 [file mmc2.docx]

Supplementary Table -1. Sequence output for each sample

| sample ID | forward sequence count | reverse sequence count |
| --- | --- | --- |
| 1 | 46728 | 46728 |
| 2 | 69183 | 69183 |
| 3 | 118838 | 118838 |
| 4 | 84595 | 84595 |
| 5 | 113772 | 113772 |
| 6 | 41989 | 41989 |
| 7 | 105761 | 105761 |
| 8 | 214670 | 214670 |
| 9 | 116080 | 116080 |
| 10 | 5230 | 5230 |
| 11 | 143836 | 143836 |
| 12 | 81211 | 81211 |
| 13 | 51509 | 51509 |
| 14 | 30432 | 30432 |
| 15 | 29332 | 29332 |
| 16 | 93462 | 93462 |
| 17 | 108075 | 108075 |
| 18 | 157949 | 157949 |
| 19 | 105788 | 105788 |
| 20 | 29699 | 29699 |
| 21 | 106844 | 106844 |
| 22 | 91922 | 91922 |
| 23 | 94721 | 94721 |
| 24 | 137810 | 137810 |
| 25 | 52953 | 52953 |
| 26 | 90965 | 90965 |
| 27 | 79783 | 79783 |
| 28 | 126077 | 126077 |
| 29 | 86445 | 86445 |
| 30 | 206036 | 206036 |
| 31 | 89927 | 89927 |
| 32 | 48848 | 48848 |
| 33 | 80997 | 80997 |
| 34 | 87052 | 87052 |
| 35 | 77978 | 77978 |
| 36 | 122033 | 122033 |
| 37 | 267448 | 267448 |
| 38 | 54316 | 54316 |
| 39 | 87375 | 87375 |
| 40 | 86700 | 86700 |
| 41 | 72989 | 72989 |
| 42 | 182792 | 182792 |
| 43 | 81574 | 81574 |
| 44 | 80603 | 80603 |
| 45 | 86889 | 86889 |
| 46 | 375996 | 375996 |
| 47 | 159017 | 159017 |
| 48 | 114386 | 114386 |
| 49 | 350940 | 350940 |
| 50 | 72914 | 72914 |
| 51 | 128904 | 128904 |
| 52 | 23967 | 23967 |
| 53 | 257585 | 257585 |

Supplementary Table-2. Ten least abundant phyla

| Phylum | Total Abundance | Percentage |
| --- | --- | --- |
| *Thermoplasmatota* | 2 | 0.00075226 |
| *SAR324_clade(Marine_group_B)* | 3 | 0.0011284 |
| *Bdellovibrionota* | 5 | 0.00188066 |
| *Acidobacteriota* | 7 | 0.00263293 |
| *Patescibacteria* | 7 | 0.00263293 |
| *Hydrogenedentes* | 11 | 0.00413745 |
| *Sumerlaeota* | 11 | 0.00413745 |
| *Fibrobacterota* | 14 | 0.00526585 |
| *Halobacterota* | 14 | 0.00526585 |
| *Elusimicrobiota* | 15 | 0.00564198 |

Supplementary Table-3. Ten least abundant genus

| Genus | Total Abundance | Percentage |
| --- | --- | --- |
| *Hydrogenispora* | 1 | 0.00037613 |
| *Staphylococcaceae* | 1 | 0.00037613 |
| *AKYH767* | 2 | 0.00075226 |
| *Acetobacterium* | 2 | 0.00075226 |
| *Acidipila* | 2 | 0.00075226 |
| *Acidothermus* | 2 | 0.00075226 |
| *Aerococcaceae* | 2 | 0.00075226 |
| *Algoriphagus* | 2 | 0.00075226 |
| *Aminobacter* | 2 | 0.00075226 |
| *Arthrospira* | 2 | 0.00075226 |

S
